# Ectopic Expression of a *Glycine soja myo*-Inositol Oxygenase Gene (*GsMIOX1a*) in *Arabidopsis* Enhances Tolerance to Alkaline Stress

**DOI:** 10.1371/journal.pone.0129998

**Published:** 2015-06-19

**Authors:** Chen Chen, Xiaoli Sun, Huizi Duanmu, Yang Yu, Ailin Liu, Jialei Xiao, Yanming Zhu

**Affiliations:** 1 Key Laboratory of Agricultural Biological Functional Genes, Northeast Agricultural University, Harbin, 150030, P.R. China; 2 Agronomy College, Heilongjiang Bayi Agricultural University, Daqing, 163319, P.R. China; Texas Tech University, UNITED STATES

## Abstract

*Myo*-inositol participates in various aspects of plant physiology, and *myo*-inositol oxygenase is the key enzyme of the *myo*-inositol oxygenation pathway. Previous studies indicated that *myo*-inositol oxygenase may play a role in plant responses to abiotic stresses. In this study, we focused on the functional characterization of *GsMIOX1a*, a remarkable alkaline stress-responsive gene of *Glycine soja* 07256, based on RNA-seq data. Using quantitative real-time PCR, we demonstrated that *GsMIOX1a* is rapidly induced by alkaline stress and expressed predominantly in flowers. We also elucidated the positive function of *GsMIOX1a* in the alkaline response in the wild type, *atmiox1* mutant as well as *GsMIOX1a*-overexpressing *Arabidopsis*. We determined that *atmiox1* mutant decreased *Arabidopsis* tolerance to alkaline stress, whereas *GsMIOX1a* overexpression increased tolerance. Moreover, the expression levels of some alkaline stress-responsive and inducible marker genes, including *H^+^-Ppase, NADP-ME, KIN1* and *RD29B*, were also up-regulated in *GsMIOX1a* overexpression lines compared with the wild type and *atmiox1* mutant. Together, these results suggest that the *GsMIOX1a* gene positively regulates plant tolerance to alkaline stress. This is the first report to demonstrate that ectopic expression of *myo*-inositol oxygenase improves alkaline tolerance in plants.

## Introduction

Soil alkalinity is one of the most serious environmental factors negatively affecting plant production in northeast China [1, 2]. Alkalization of soil due to NaHCO_3_ and Na_2_CO_3_ can be more destructive than soil salinization caused by neutral salts, such as NaCl and Na_2_SO_4_ [3]. Alkaline stress not only involves ion injury, excessive levels of reactive oxygen species (ROS) and osmotic stress [4, 5] but also acts as a high-pH stressor. A high-pH environment surrounding roots may inhibit ion uptake and disrupt ion homeostasis in plant cells [6]. Consequently, growth and photosynthesis are negatively affected [7]. However, to date, tolerance to alkaline stress has not been intensively studied in plants. *Glycine soja* 07256 (*G*. *soja* 07256) can germinate and grow in soil at pH 9.02 and survive in nutrient solutions containing 50 mM NaHCO_3_ [8], indicating that this plant has developed unique molecular and physiological mechanisms to adapt to these stress conditions. Therefore, *G*. *soja* 07256 is an ideal candidate for screening stress-resistance genes and studying the molecular mechanisms of plant stress tolerance.

*Myo*-inositol oxygenase (MIOX; E.C. 1.13.99.1) is a monooxygenase that catalyzes the conversion of *myo*-inositol into D-glucuronic acid (D-GlcUA). MIOX proteins are highly conserved and present in nearly all eukaryotes. Many animals, including rats, use this product to synthesize ascorbic acid (AsA). MIOX first degrades *myo*-inositol to D-GlcUA. This step is followed by reduction to L-gulonic acid and ring formation to gulonolactone, which is finally oxidized to AsA [9]. Plants have also established the pathway for biosynthesizing D-GlcUA with MIOX activity as the committed step, whereas plants further use this product and activate it into UDP-glucuronic acid (UDP-GlcUA). UDP-GlcUA is an important precursor for several nucleotide sugars that are used to synthesize cell wall polysaccharides. The finding was supported by ^3^H-myoinositol labeling studies [10, 11] as well as the analysis of knockout mutants in *Miox1* and *Miox2* of *Arabidopsis* [12, 13]. Therefore, one of the important functions of MIOX involves controlling the level of *myo*-inositol, which is a precursor for many inositol-containing compounds that are involved in various physiological and biochemical processes, such as growth regulation, cell membrane biogenesis, hormonal regulation and stress signaling [14]. MIOX studies in plants were previously limited because the content of MIOX was difficult to measure in plant extracts and no molecular sequences were available. Recently, peptide sequences were obtained by purifying the enzymes from pig kidneys [15] and the yeast *Cryptococcus lactativorus* [16], thereby facilitating the subsequent cloning of the corresponding genes. With the exception of the D-mannose/L-galactose (Man/Gal), D-galacturonate (GalU) and L-gulose pathways, an animal-like route for AsA synthesis in plants has been identified [17]. Some studies have suggested that MIOX gene overexpression leads to a 2- to 3-fold increase in AsA in transgenic *Arabidopsis* lines [18]; Zhang et al. demonstrated that overexpression of a purple acid phosphatase resulted in an increase in D-GlcUA in *Arabidopsis* [19]. Because the product of the MIOX reaction was D-GlcUA, their data suggested that this metabolite is readily converted to AsA in *Arabidopsis*, resembling the pathway characterized in animals. However, recent studies reported that MIOX controls the level of *myo*-inositol without increasing AsA content [20, 21], which was also confirmed by J. Duan et al. in a study reporting that *OsMIOX* (*Oryza sativa* L. cv. IRAT109) overexpression improves drought tolerance in rice, but the level of AsA was not altered before and after stress [22]. Therefore, whether MIOX has a positive role in AsA biosynthesis in plants remains unclear.

We analyzed RNA-seq data from *G*. *soja* 07256 treated with 50 mM NaHCO_3_ (pH 8.5) and observed that *GsMIOX1a* was among those genes most strongly up-regulated (raw sequence data are available at http://www.onekp.com/samples/single.php?id=LXGM). In this study, we aimed to identify the functional and regulatory roles of *GsMIOX1a* in plant responses to alkaline stress. We analyzed alkaline stress-induced transcript accumulation of *GsMIOX1a* in a time-dependent manner as well as the expression profiles of *GsMIOX1a* in various organs of *G*. *soja* 07256. To verify the positive role of *GsMIOX1a* in the alkaline response, we functionally validated gene expression under conditions of alkaline stress in wild type (WT), *GsMIOX1a*-overexpressing (OX) *Arabidopsis* and an *Arabidopsis* mutant with a T-DNA insertion of a homologous gene, *atmiox1*. Furthermore, we assessed the physiological mechanism by which *GsMIOX1a* OX lines exhibit increased alkaline tolerance. Collectively, these results provide direct evidence that *GsMIOX1a* positively regulates the plant alkaline stress response and will facilitate further studies of the biological functions and molecular mechanisms of plant MIOX in response to abiotic stresses.

## Materials and Methods

### Plant materials and stress treatments

Seeds of *G*. *soja* 07256 and *G*. *soja* 50109 were obtained from the Jilin Academy of Agricultural Sciences (Changchun, China). *G*. *max* Suinong 28 and *G*. *max* Hefeng 55 were obtained from the Chinese Crop Germplasm Information System.

To analyze gene transcript accumulation induced by alkaline stress, after treatment with 98% sulfuric acid for 10 min, the seeds of *G*. *soja* 07256 were washed five times with sterilized water and then germinated and grown in 1/4 Hoagland solution for 3 weeks at 24 to 26°C and a 16-h light:8-h dark cycle. The roots of 21-day-old seedlings were submerged in 1/4 Hoagland solution containing 50 mM NaHCO_3_ (pH 8.5). Samples of roots were harvested at 0, 1, 3, 6, 12 and 24 h after treatment. Then, the samples were frozen in liquid nitrogen and stored at -80°C for RNA extraction.

For *GsMIOX1a* expression analysis in different soybean varieties, seeds of *G*. *soja* 07256, *G*. *soja* 50109, *G*. *max* Suinong 28 and *G*. *max* Hefeng 55 were placed on each petri dish with wet filter paper to accelerate germination for 2 days. Germinated seedlings were then transferred into 1/4 Hoagland solution. Three weeks after sowing, seedlings were transferred into 1/4 Hoagland solution with 50 mM NaHCO_3_. Equal amounts ofroots were sampled at 0 and 6 h.

Wild type *Arabidopsis thaliana* (Columbia ecotype) and the *atmiox1* mutant (SALK_082070) were obtained from the Nottingham Arabidopsis Stock Centre (NASC). *Arabidopsis* seeds were germinated and grown on 1/2 MS media with 0.8% agar or in pots filled with a 1:1:1 mixture of vermiculite: peat moss: perlite in a growth chamber under controlled environment conditions (21 to 23°C, 16-h light:8-h dark cycle).

### *GsMIOX1a* cDNA cloning

The full-length *GsMIOX1a* gene was obtained via its homologous gene in *G*. *max* (Glyma07g01660). Briefly, total RNA was extracted from 21-day-old *G*. *soja* 07256 seedlings using an RNAprep Pure Plant Kit (Tiangen, China). Then, the cDNA was generated with a SuperScript III Reverse Transcriptase Kit (Invitrogen, Carlsbad, CA, USA). Gene-specific primers, *GsMIOX1*a-F: 5’-ATGACTATCCTCATTGAGCAATCTGATC-3’ and *GsMIOX1*a-R: 5’-TCACCACTTCAGCTTTGCAGGGA-3’, were designed according to the corresponding gene sequence from *G*. *max*, and the full-length CDS region of *GsMIOX1a* was obtained by polymerase chain reaction (PCR) amplification. The PCR products were cloned into the pGEM-T cloning vector (Promega, Madison, WI, USA), and the resulting plasmids were sequenced.

### Quantitative real-time PCR analysis

Total RNA extraction from different tissues and cDNA synthesis were performed as described above. Quantitative real-time PCR was performed on a Stratagene MX3000p real-time cycler using SYBR Green. One reaction (10 μL) consisted of 1 μL of cDNA (diluted 1: 5), 5 μL of SYBR Green Real-time PCR Master Mix (TOYOBO, USA), 400 nM of each primer, and 3.2 μL of ddH_2_O. The housekeeping genes *AtACTIN2* (F: 5’-TTACCCGATGGGCAAGTC-3’; R: 5’-GCTCATACGGTCAGCGATAC-3’) and *GsGAPDH* (glyceraldehyde 3-phosphate dehydrogenase; F: 5’-GACTGGTATGGCATTCCGTGT-3’; R: 5’-GCCCTCTGATTCCTCCTTGA -3’) were used for internal normalization of *Arabidopsis* and *G*. *soja* genes, respectively [23]. For calculations, the reaction efficiency of the individual well was computed from the original data. Results were averaged over three triplicates and normalized using the calculated levels of *AtACTIN2* or *GsGAPDH* transcripts. The relative intensities were calculated and normalized as previously described [24].

Primers used in determining the expression levels of alkaline stress-responsive and inducible marker genes in WT, *atmiox1* and *GsMIOX1a* OX plants were: *H* ^*+*^-*Ppase*-F: 5’-ATGACGATGATGAAGAAGAAGAAGAT-3’ and *H* ^*+*^-*Ppase*-R: 5’-TTTTTTAACCACCTACGGTAAACG-3’; *RD29B*-F: 5’-GCGCACCAGTGTATGAATCC TC-3’ and *RD29B*-R: 5’-TGTGGTCAGAAGACACGACAGG-3’; *NADP-ME*-F: 5’-TGGTCTGATCTACCCG CCATT-3’ and *NADP-ME*-R: 5’-CGCCAATCCGAGGTCATAGG-3’; *KIN1*-F: 5’-AACAAGAATGCCTTCCA AGC-3’ and *KIN1*-R: 5’- CGCATCCGATACACTCTTTCC-3’.

### Characterization of the *atmiox1* mutant

PCR and semi-quantitative reverse transcriptase (RT)-PCR analyses were performed to characterize the *AtMIOX1* gene T-DNA insertion mutant of *Arabidopsis* (*atmiox1*, SALK_082070). First, the forward (FP: 5’-GTGAAACCCTACAAGCAGAAAGC-3’) and reverse (RP: 5’-CGATTTCAAGCCGTCCACA-3’) primer pair was used to determine if the T-DNA insertion was homozygous, and the FP and left border (LB: 5’-GATTTGGGTGATGGTTCACGTAGTG-3’) primer pair was used to verify that the T-DNA was inserted into the *AtMIOX1* gene. In addition, semi-quantitative RT-PCR using the FP and RP primer pair was performed to further identify whether the *AtMIOX1* gene was efficiently silenced by the T-DNA insertion in the *atmiox1* mutant. Briefly, total RNA was extracted from 2-week-old WT and *atmiox1* seedlings and then reverse transcribed to generate cDNA. The yield of cDNA was normalized according to the PCR signal generated from the internal standard housekeeping gene *ACTIN2* amplified with 25 cycles starting with 0.2 μl of the cDNA solution. *AtMIOX1* gene expression levels were analyzed by PCR amplification for 30 cycles.

### Generation of *GsMIOX1a* OX *Arabidopsis thaliana* plants

To generate *GsMIOX1a* OX lines, the complete coding region of *GsMIOX1a* was cloned into the pCAMBIA330035Su vector as described [25] by using primers F: 5’-GGCTTAAUATGACTATCCTCATTGAG CAA-3’ and R: 5’-GGTTTAAUTCACCACTTCAGTTTGC-3’ and introduced into the *Agrobacterium tumefaciens* strain GV3101. Then, the floral dip method was used to transform *Arabidopsis thaliana* [26]. The T_0_ generation seeds were grown on selective solid medium containing 50 mg/L glufosinate ammonium (Sigma-Aldrich), and positive transformations were confirmed by PCR using primers designed based on the coding DNA sequence (CDS) region of *GsMIOX1a*. Homozygous OX lines from the T_3_ generation that displayed 100% resistance to glufosinate ammonium were confirmed by semi-quantitative RT-PCR analysis as described above. Primer pair was *GsMIOX1a*-F: AGGCGTGAAGGAGTGGAGAAC-3’ and *GsMIOX1a*-R: GGATAAGGCCAGTTAAGTGCAA-3’ and the housekeeping gene *ACTIN2* served as an internal control. PCR conditions were 30 cycles for *GsMIOX1a* and 25 cycles for *ACTIN2*.

### Phenotype analysis of the *GsMIOX1a* OX and *atmiox1* mutant lines

Seeds from the WT, *atmiox1* and *GsMIOX1a* OX lines were surface sterilized with 5% (w: v) sodium hypochlorite for 7 min and subsequently washed with sterilized distilled water 6 to 8 times. The seeds were then stored in the dark at 4°C for 3 days to break seed dormancy. In plate germination assays, the seeds of the WT, *atmiox1* and *GsMIOX1a* OX lines (independent lines #4, #16, and #20) were germinated on 1/2 MS agar medium supplemented with 11 mM NaHCO_3_. The germination profiles were observed with respect to radicle emergence for 7 consecutive days. On the 3rd day, photographs were taken to demonstrate the growth performance of each line. In total, 120 seeds were used for each experiment, and all experiments were repeated at least three times. For soil alkaline stress treatment, three-week-old WT, *atmiox1* and *GsMIOX1a* OX seedlings were irrigated with 100 mM NaHCO_3_ (pH 8.5) every 3 days for a total of 12 days for alkaline stress.

To measure the free proline concentration, peroxidase (POD) activity and AsA content, leaf samples were obtained from 5-week-old plants under control conditions as well as 100 mM NaHCO_3_ treatment. For free proline measurement, leaves (approximately 0.25 g) were extracted with 5 mL of 3% sulfosalicylic acid, and measurements were obtained using the ninhydrin assay [27]. The proline concentration was estimated at 520 nm using a spectrophotometer (UV-2550, Shimadzu, Japan). For POD, the oxidation of guaiacol was determined by measuring the increased absorbance values at 470 nm for 60 s (linear phase) [28]. The 1.8 mL reaction mixture contained 1.45 mL of 0.05 mmol·L^-1^ phosphate buffer (pH 7.0), 0.05 mL of 2% guaiacol, 0.05 mL of enzyme extract and 0.25 mL of 2% H_2_O_2_. A unit of enzyme activity was defined as the change in the absorbance value per minute per mg fresh weight. The AsA content was measured as described by Arakawa et al [29]. Approximately 0.30 g of leaves was frozen in liquid nitrogen, homogenized in 6 mL of 5% trichloroacetic acid (TCA) solution, and centrifuged at 15,000×g for 15 min. The supernatant was used to measure the AsA content using 0.5% 4, 7-diphenyl-1, 10-phenanthroline.

## Results

### Isolation and bioinformatic analysis of the *GsMIOX1a* gene

**Fig 1:**
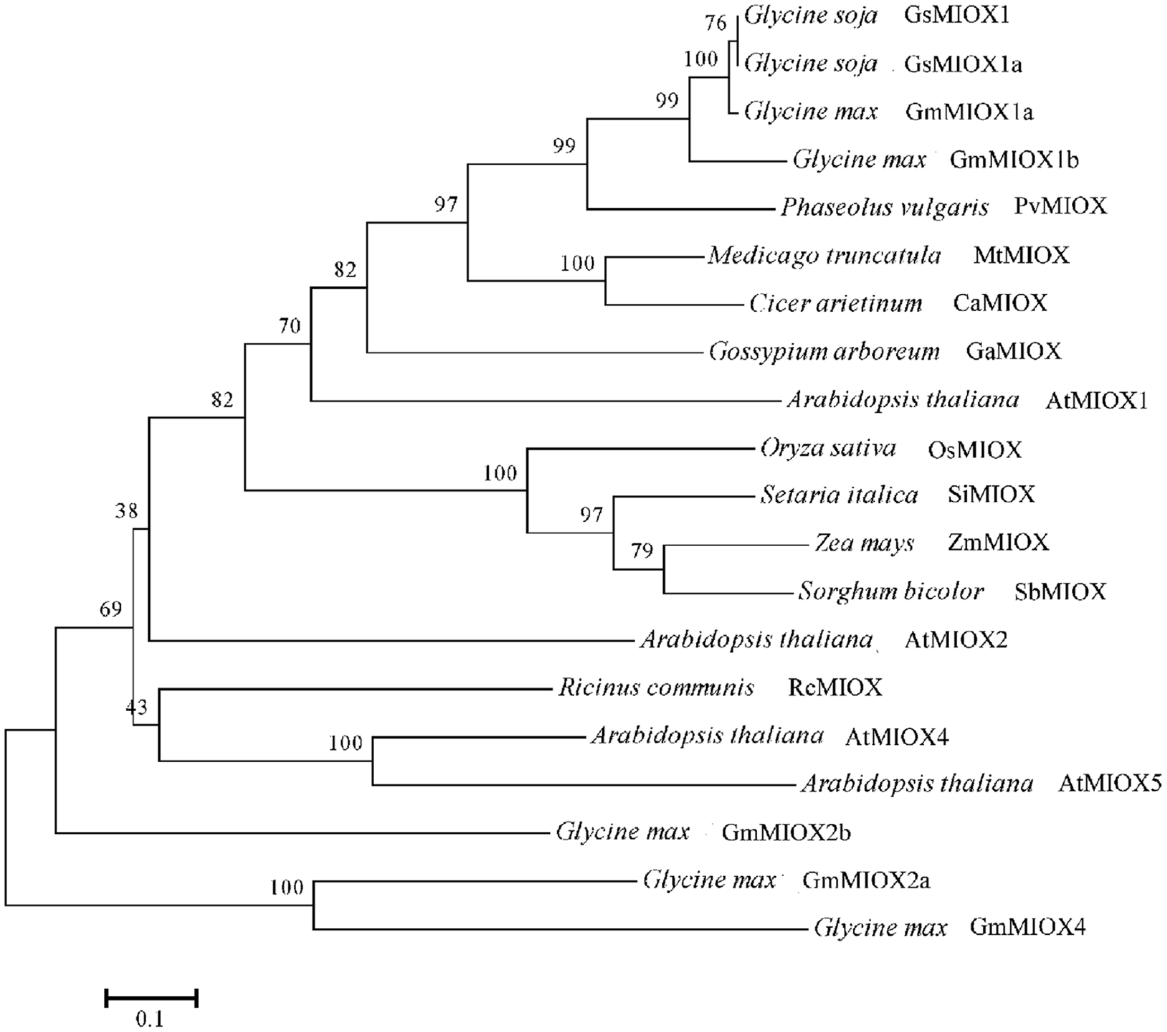
The phylogenetic tree of GsMIOX1a and MIOX from different plants. The analysis was based on a multiple amino acid sequence alignment. A neighbor-joining tree was generated using MEGA 5.0. The aligned protein sequences from GenBank (with accession numbers) included GsMIOX1 (*Glycine soja*, KHN39268), AtMIOX1 (*Arabidopsis thaliana*, NP_001154337), AtMIOX2 (*Arabidopsis thaliana*, NP_565459), AtMIOX4 (*Arabidopsis thaliana*, NP_194356), AtMIOX5 (*Arabidopsis thaliana*, NP_200475), MtMIOX (*Medicago truncatula*, KEH18252), CaMIOX (*Cicer arietinum*, XP_004510326), OsMIOX (*Oryza sativa*, NP_001057871), ZmMIOX (*Zea mays*, NP_001141330), PvMIOX (*Phaseolus vulgaris*, XP_007135612), GaMIOX (*Gossypium arboretum*, KHF98793), SiMIOX (*Setaria italica*, XP_004966088), GmMIOX1a (*Glycine max*, Glyma07g01660), GmMIOX1b (*Glycine max*, Glyma08g21300), GmMIOX2a (*Glycine max*, Glyma01g00840), GmMIOX2b (*Glycine max*, Glyma07g15190), GmMIOX4 (*Glycine max*, Glyma08g01690), SbMIOX (*Sorghum bicolor*, Sb10g022160), and RcMIOX (*Ricinus communis*, 29912.m005347) with gene ID from Phytozome v9.1.

*GsMIOX1a* was isolated using homology-based cloning. The 921-bp coding sequence encodes a 306-amino acid protein that corresponds to a polypeptide with a predicted molecular weight (MW) of 35.6 kDa. The deduced protein sequence of *GsMIOX1a* was used as a query for a BLASTP search against the *G*. *max* protein database Phytozome v9.1 (http://www.phytozome.net/). From this search, we identified 5 putative MIOX protein sequences in soybean, and these genes were named according to the existing numbering system used in the phylogenetic tree of *Arabidopsis*. The characteristics of the *G*. *max* and *Arabidopsis* MIOX family genes, including the gene ID, full CDS length, genomic length, protein length, molecular weight and isoelectric point (pI), are presented in Table 1. Furthermore, we aligned the *GsMIOX1a* amino acid sequence with the MIOX amino acid sequences from other species (S1 Fig). The analysis revealed a high degree of sequence identity, including 100% identity with *GsMIOX1* (KHN39268). Because the *GsMIOX1* coding sequence was unavailable, we hypothesized that these sequences might represent the same gene. Then, a phylogenetic tree was constructed based on the above sequences using MEGA 5.0. The phylogenetic tree indicated that the amino acid sequences of monocot MIOX1 and dicot MIOX1 were in separate clusters. *GsMIOX1a* appeared in the dicot cluster and exhibited a closer relationship to the *MIOX1* genes from other plants than to *MIOX2*, *MIOX4* or *MIOX5* from other species (Fig 1).

**Table 1:** Basic information on the MIOX family in *Arabidopsis thaliana* and *G*. *max*.

Gene name	Gene ID	Genomic length (bp)	Full CDS length (bp)	Protein length (aa)	Molecular weight (Da)	pI
*AtMIOX1*	AT1G14520	2170	936	311	36573.75	4.93
*AtMIOX2*	AT2G19800	2613	954	317	37047.88	5.34
*AtMIOX4*	AT4G26260	2517	957	318	36903.52	4.89
*AtMIOX5*	AT5G56640	2315	945	314	36544.13	5.22
*GmMIOX1a*	Glyma07g01660	4087	936	311	36582.30	5.26
*GmMIOX1b*	Glyma08g21300	4486	921	306	36147.73	5.14
*GmMIOX2a*	Glyma01g00840	2949	936	311	36106.60	5.16
*GmMIOX2b*	Glyma07g15190	4847	951	316	29859.86	6.72
*GmMIOX4*	Glyma08g10690	2231	783	260	36667.32	5.18

### *GsMIOX1a* expression is alkaline stress inducible and tissue specific

**Fig 2:**
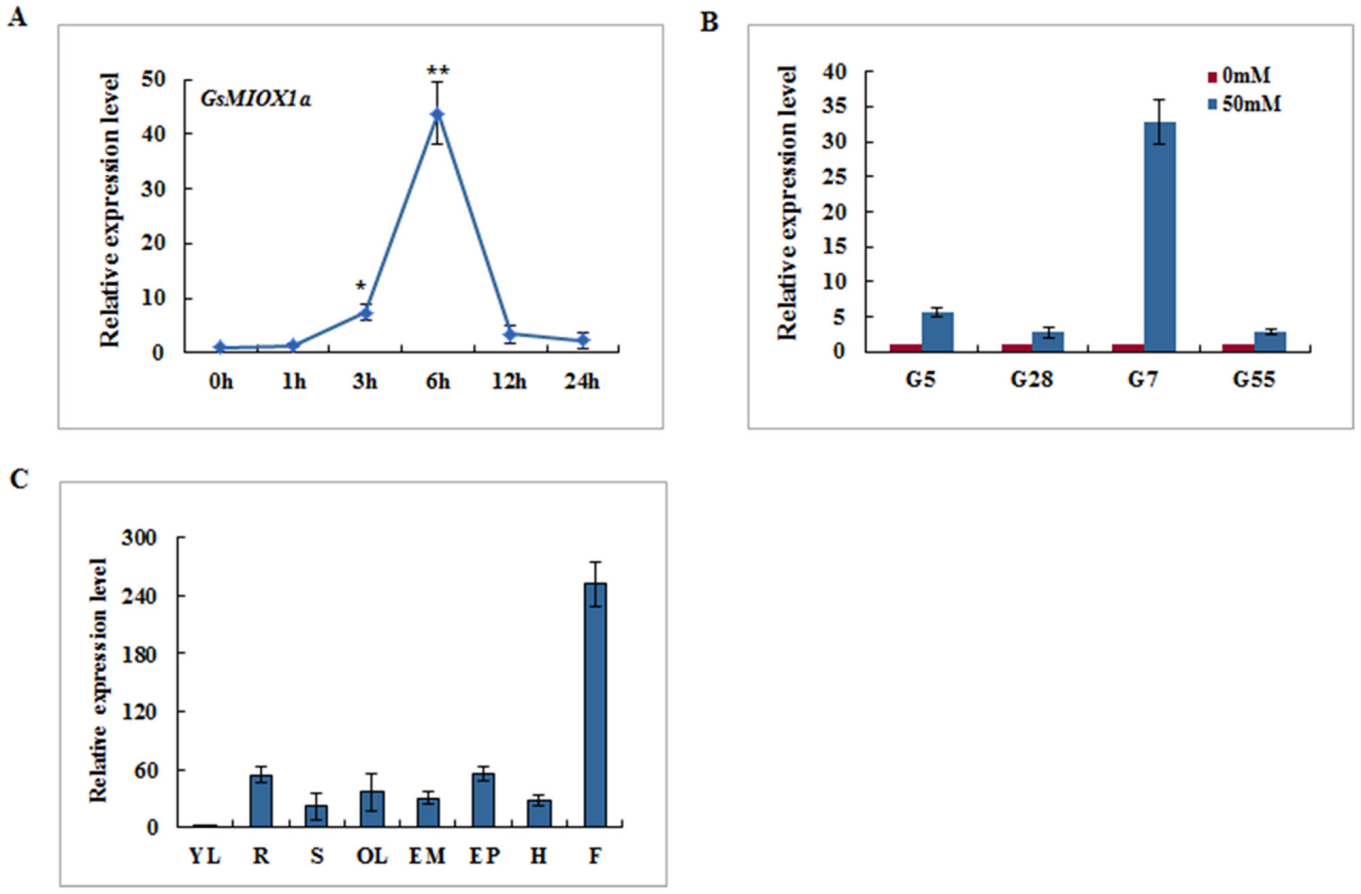
Expression patterns of *GsMIOX1a* in *G*. *soja* 07256. (A) The accumulation of *GsMIOX1a* transcripts under 50 mM NaHCO_3_ (pH 8.5) in *G*. *soja* 07256 as determined by quantitative real-time PCR analysis. Root samples were collected at 0, 1, 3, 6, 12, and 24 h. (B) Expression levels of *GsMIOX1a* in different soybean varieties including *G*. *soja* 50109 (G5), *G*. *max* Suinong 28 (G28), *G*. *soja* 07256 (G7), and *G*. *max* Hefeng 55 (G55). Total RNA was extracted from the roots of 3-week-old seedlings of four soybean varieties whose roots had been submerged in nutrient solution containing 50 mM NaHCO_3_. (C) The tissue-specific expression analysis of *GsMIOX1a* as determined by quantitative real-time PCR. The tissues included young leaf (YL), root (R), stem (S), old leaf (OL), episperm (EM), epicotyl (EP), hypocotyl (H) and flower (F). *GAPDH* was used as an internal control. The experiment included three fully independent biological repeats, and three technical repeats and the mean value are presented. The error bar represents the ± S.E. (standard error, n = 3). Significant differences analyses were conducted using the T-test method and are denoted by one or two stars, corresponding to p<0.05 and p<0.01, respectively.

To explore the biological function of *GsMIOX1a* in response to alkaline stress, we initially investigated *GsMIOX1a* transcript accumulation under alkaline stress by quantitative real-time PCR analysis. As shown in Fig 2A, under alkaline stress, *GsMIOX1a* expression levels increased and reached a maximum level at 6 h that was approximately 42-fold higher than that at 0 h. This result was consistent with our previous RNA-seq data (Figure A in S1 File), which also strongly suggested a role of *GsMIOX1a* in the alkaline stress response.

Because we screened *GsMIOX1a* as a putative gene from *G*. *soja* 07256 involved in the response to alkaline stress, we further investigated the *GsMIOX1a* response to alkaline stress in other soybean varieties, including *G*. *soja* 07256, *G*. *soja* 50109, *G*. *max* Suinong 28 and *G*. *max* Hefeng 55. *G*. *ma*x Suinong 28 and *G*. *max* Hefeng 55 are Chinese soybean cultivars that exhibit much lower adaptability to stress compared to wild soybean. *G*. *soja* 50109 was also a kind of wild soybean but didn’t reported to be alkaline resistant. We determined the transcript expression levels of *GsMIOX1a* using roots of four soybean variety seedlings under 50 mM NaHCO_3_ treatment by quantitative real-time PCR (Fig 2B). Under alkaline stress, the relative transcript abundance of *GsMIOX1a* increased by 32.8-fold in *G*. *soja* 07256 and 5.7-fold in *G*. *soja* 50109. By contrast, the expression of *GsMIOX1a* was relatively unchanged in *G*. *max* Suinong 28 and *G*. *max* Heheng 55, which exhibit reduced tolerance to alkaline stress. This result suggests that *GsMIOX1a* is an important responsive gene in alkaline-resistant soybean varieties.

As a step toward elucidating *GsMIOX1a* gene function in *G*. *soja* 07256, we surveyed *GsMIOX1a* expression profiles across different organs using quantitative real-time PCR. *G*. *soja* 07256 seedlings were planted and maintained under normal conditions, and total RNA was isolated from different organs. As shown in Fig 2C, the *GsMIOX1a* transcript was detected in roots, stems, old leaves, episperms, epicotyls, hypocotyls and flowers; however, minimal *GsMIOX1a* transcript was observed in young leaves. Among these examined tissues, increased *GsMIOX1a* transcript levels accumulated in flowers, as determined by quantitative real-time PCR. The quantitative real-time PCR results were confirmed by three independent experiments.

### Characterization of the *GsMIOX1a* OX and *atmiox1* mutant lines

**Fig 3:**
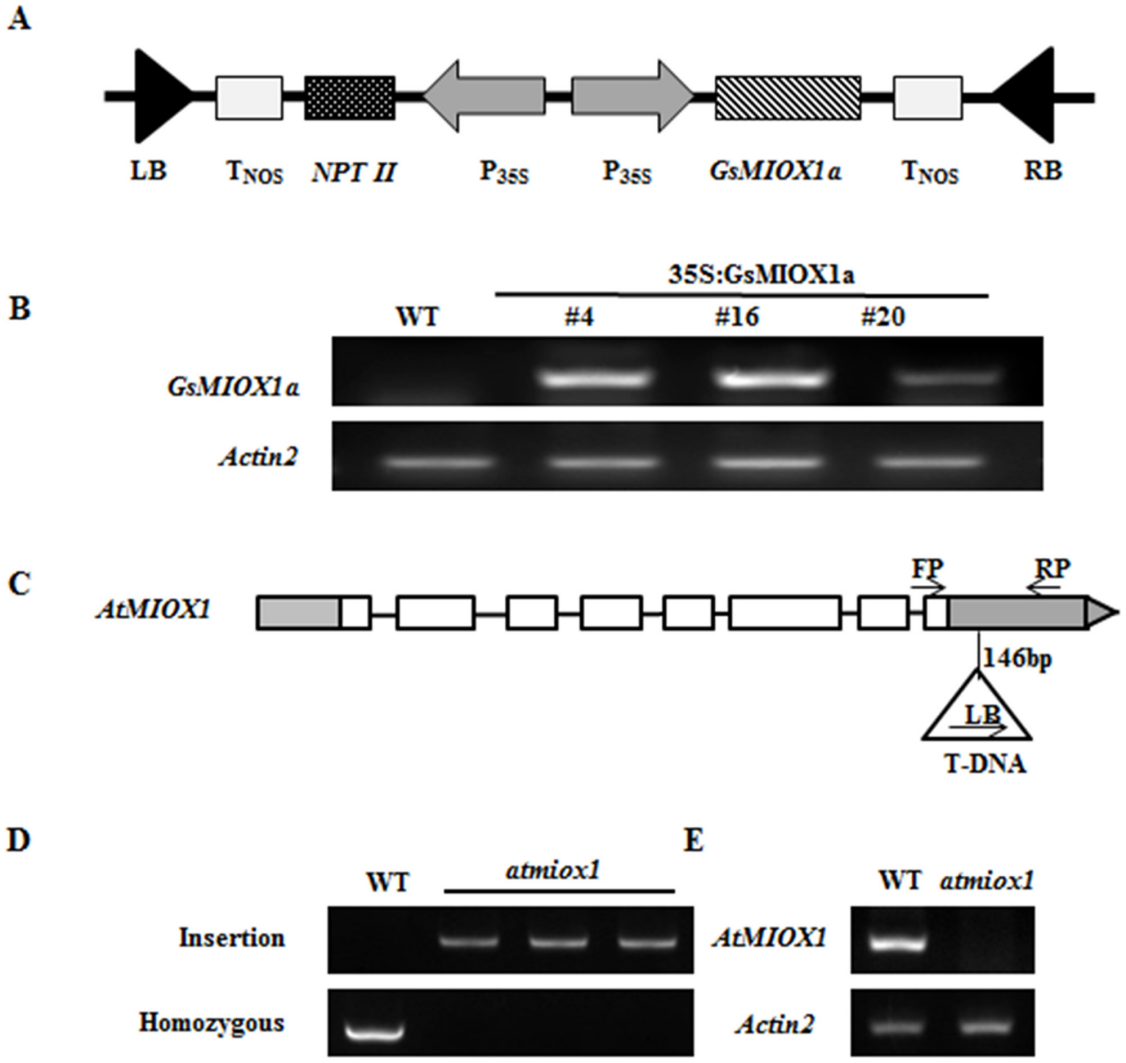
Characterization of the *GsMIOX1a* OX and *atmiox1* mutant lines. (A) Schematic representation of the construct for *GsMIOX1a* gene overexpression in *Arabidopsis*. (B) *GsMIOX1a* gene expression in the WT and OX lines. The expression levels were analyzed by semi-quantitative RT-PCR, and the *ACTIN2* gene was used as an internal standard. (C) T-DNA and the flanking sequence of the *AtMIOX1* gene in the *atmiox1* mutant. The FP and RP primer pair was used for homozygous analysis, and the LB and RP primer pair was used for T-DNA insertion analysis. (D) PCR identification of the *atmiox1* mutant. (E) Semi-quantitative RT-PCR identification of the *atmiox1* mutant.

To clarify the regulatory role of *GsMIOX1a* gene in *Arabidopsis*, we used constitutive OX lines in which expression was controlled by the strong constitutive CaMV35S promoter (Fig 3A). T_0_ generation *GsMIOX1a* transformed plants were initially screened for glufosinate ammonium resistance, and T_1_ generation OX lines, which displayed a 3:1 (resistant: sensitive) segregation pattern in their progeny, were selected for further analysis. Three homozygous T_3_ OX *Arabidopsis* lines (#4, #16, #20) as determined by PCR that were 100% resistant to glufosinate ammonium were obtained. Semi-quantitative RT-PCR analysis confirmed increased *GsMIOX1a* transcript levels in the OX lines compared with WT plants (Fig 3B). We also examined an *AtMIOX1* gene knockout mutant in *Arabidopsis*, *atmiox1*, carrying a T-DNA insertion in the 3’UTR located 146 bp downstream of the last exon (Fig 3C). We verified the genotype of mutant plants by PCR tests using the FP and RP primers for the homozygous analysis and the LB and RP primers for the T-DNA insertion analysis. As shown in Fig 3D (top lane), when assessing the T-DNA insertion using the LB and RP primers, no clear DNA band was observed for WT, but a clear DNA band was observed for the T-DNA insertion in the mutant. By contrast, no DNA band appeared for *atmiox1* when we performed the PCR test with the FP and RP primers for the homozygous analysis. These results indicated that the T-DNA insertion of the *atmiox1* mutant was homozygous. To verify that the *AtMIOX1* gene was efficiently silenced in the *atmiox1* line, we assessed the expression of *AtMIOX1* using semi-quantitative RT-PCR analysis. In contrast to WT *Arabidopsis*, no *AtMIOX1* transcripts were detected in the *atmiox1* line (Fig 3E), indicating that *AtMIOX1* was effectively silenced in the *atmiox1* line.

### *GsMIOX1a* positively regulates plant tolerance to alkaline stress

**Fig 4:**
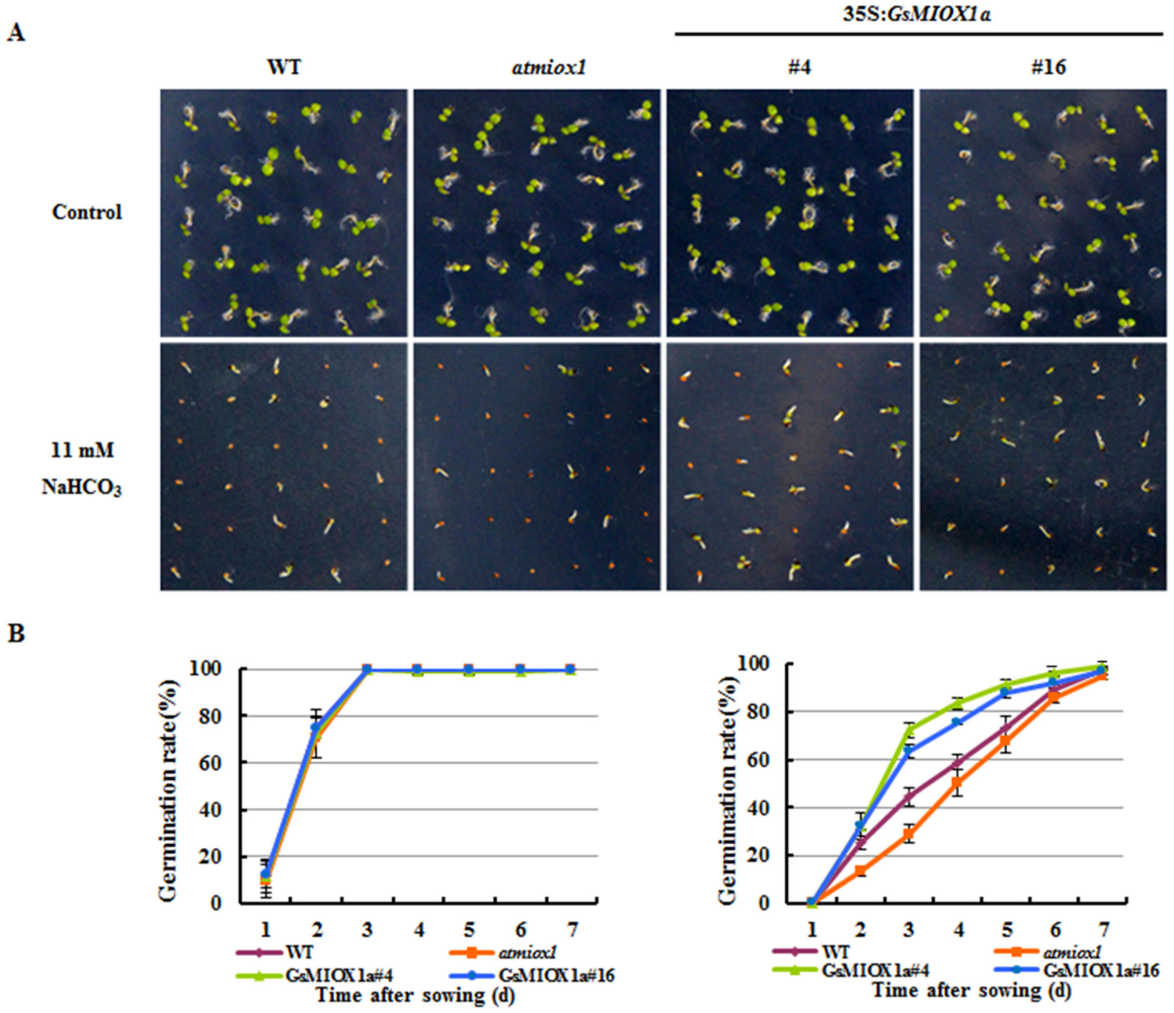
Effect of alkaline stress on the germination of the WT, *atmiox1*, and *GsMIOX1a* OX lines. (A) Phenotypes of WT, *atmiox1* and OX plants grown on 1/2 MS medium with or without 11 mM NaHCO_3_. Photographs were taken 3 days after germination. (B) The seed germination rates of the WT, *atmiox1* and OX *Arabidopsis* lines. Seeds were considered to be germinated when the radicles completely penetrated the seed coats. A total of 120 seeds from each line were used for each experiment.

To investigate the possible role of *GsMIOX1a* in plant stress adaptation and determine whether *GsMIOX1a* improves the tolerance of the plant to alkaline stress, the phenotypes of the WT, *atmiox1* and OX *Arabidopsis* were evaluated. WT, *atmiox1* and two independent *GsMIOX1a* OX lines (#4, #16) with similar *GsMIOX1a* transcript accumulation levels were chosen. Initially, seeds were planted in 1/2 MS solid medium, and no significant difference in the germination rate was observed among WT, *atmiox1* or *GsMIOX1a* OX lines. However, radicle emergence from the seeds of *atmiox1* was inhibited more severely, whereas the *GsMIOX1a* OX lines performed better than WT lines in 1/2 MS solid medium under alkaline stress (Fig 4A). When treated with 11 mM NaHCO_3_, the germination rates of *GsMIOX1a* OX seeds were maintained at relatively high levels (#4, 70% and #16, 65.5%) on the 3rd day compared with WT (44.1%), but the germination rate of the *atmiox1* seeds was reduced to 27.4% (Fig 4B). These results suggested that the alkaline tolerance of *atmiox1* was decreased at the seed germination stage, whereas tolerance increased in the *GsMIOX1a* OX lines. In addition, these results further confirmed the positive role of *GsMIOX1a* in the alkaline stress response.

**Fig 5:**
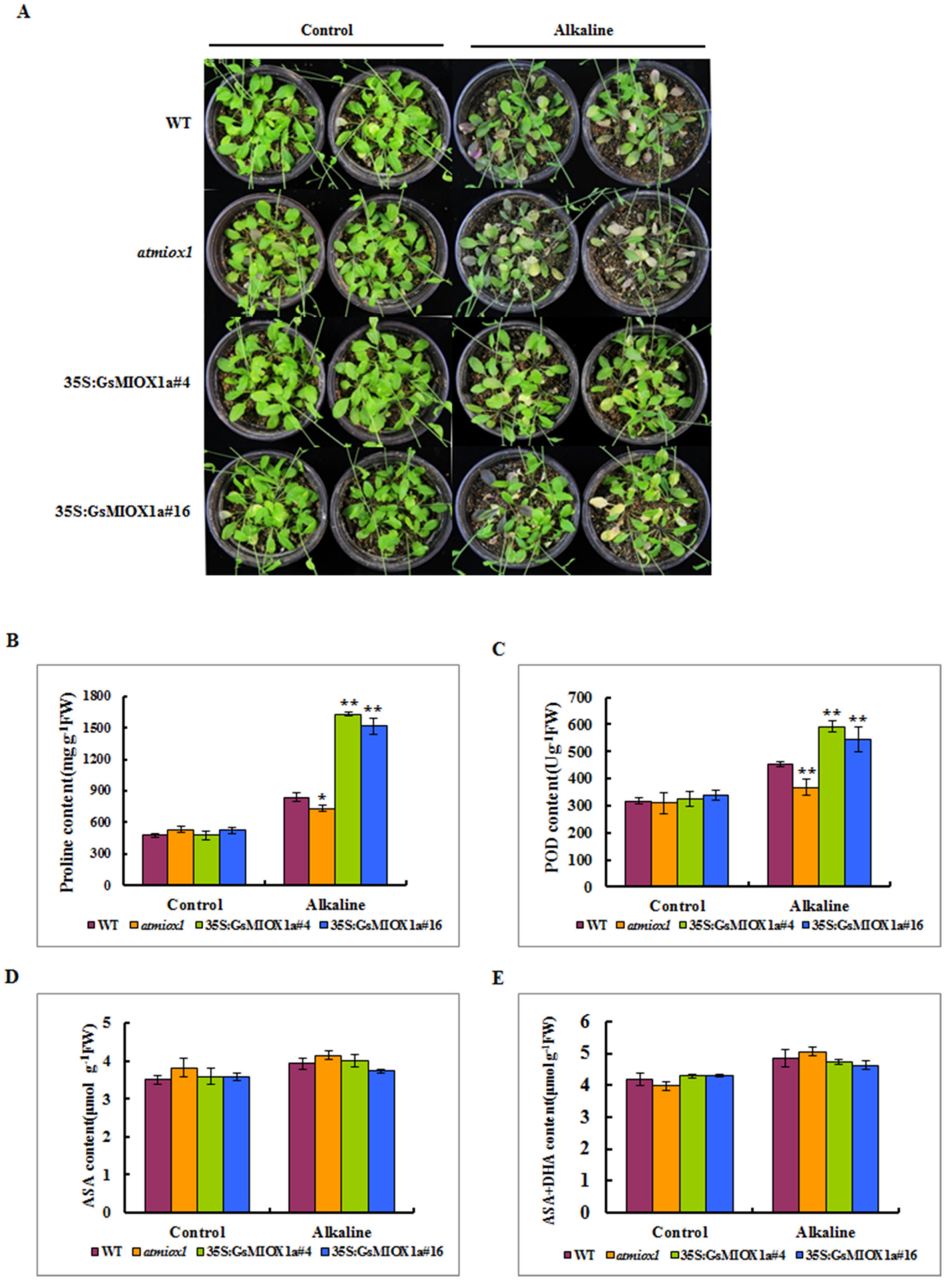
Effect of alkaline stress on mature plants of the WT, *atmiox1*, and *GsMIOX1a* OX lines. (A) Phenotypes of the WT, *atmiox1* and OX plants in response to 100 mM NaHCO_3_ stress. Three-week-old *Arabidopsis* plants were irrigated with 100 mM NaHCO_3_ solution every 3 days for a total of 12 days. The photographs were taken on the 13th day after stress exposure. (B) The free proline content of the WT, *atmiox1* and OX plants under control conditions or stress treatment. (C) The POD activity of the WT, *atmiox1* and OX plants under control conditions or alkaline stress. (D-E) The AsA content in the leaf extracts of the *GsMIOX1a* OX *Arabidopsis*, *atmiox1* and WT lines. The total amount of AsA comprised the reduced (AsA) and oxidized forms (DHA). AsA contents were measured in the leaves of 5-week-old *GsMIOX1a* OX *Arabidopsis*, *atmiox1* and WT plants under normal and alkaline stress conditions in a growth chamber. Leaves of the same age and size were selected to minimize experimental error. Error bars represent the ± S.E. (n = 3). Significant differences were analyses using the T-test method. Significant differences from WT are denoted by one or two stars, corresponding to p<0.05 and p<0.01, respectively.

We also investigated the growth pattern of soil-grown mature plants under alkaline stress. The seeds were planted in pots and allowed to grow normally for three weeks with routine watering. Then, the 3-week-old plants were irrigated with 100 mM NaHCO_3_ solution every three days for 12 days, and the growth pattern was monitored. The WT plants developed chlorotic symptoms and became yellow and purple, whereas the *atmiox1* plants exhibited more severe symptoms and began to die. By contrast, the *GsMIOX1a* OX lines remained green and survived better than control plants (Fig 5A). Several stress-related physiological and biochemical parameters were also assessed in WT, *atmiox1* and OX plants. Alkaline conditions inhibit plant growth due to a high pH and increased ROS and osmotic stress [30]. POD is one of the most important scavenging enzymes in the ROS-scavenging systems of plants [31]. Free proline, an osmoprotectant, aids in reducing the detrimental effects of alkaline conditions on cellular components [32]. Therefore, free proline concentrations along with POD activity under alkaline stress treatment were measured to understand the physiological mechanisms responsible for the increased alkaline tolerance of the *GsMIOX1a* OX lines and decreased alkaline tolerance of the *atmiox1* line. As shown in Fig 5B, after prolonged exposure to alkaline stress, the proline content was increased in all lines with respect to normally grown seedlings. However, *GsMIOX1a* OX plants exhibited increased proline levels compared with WT plants (p<0.01), whereas *atmiox1* mutant exhibited decreased total free proline (p<0.05). POD activity was increased in *GsMIOX1a* OX lines grown in stress conditions compared with WT plants (p<0.01), and POD activity in *atmiox1* mutant plants was lower than WT plants (p<0.01). POD activity appeared to be less inhibited in the *GsMIOX1a* OX lines under alkaline stress conditions; therefore, *GsMIOX1a* OX lines retained more activity than the WT or *atmiox1* mutant lines (Fig 5C).

In addition, previous studies have proposed a role for the MIOX pathway in the synthesis of AsA [33]. To determine if the insensitivity of the *GsMIOX1a* OX lines was due to a change in AsA levels, we also measured the AsA content in the WT, *atmiox1* and OX plants. However, no significant difference was observed in AsA levels among WT, *atmiox1* and OX plants under normal and alkaline stress conditions (Fig 5D and 5E).

### Alkaline response and stress-inducible marker genes are affected by *GsMIOX1a*

**Fig 6:**
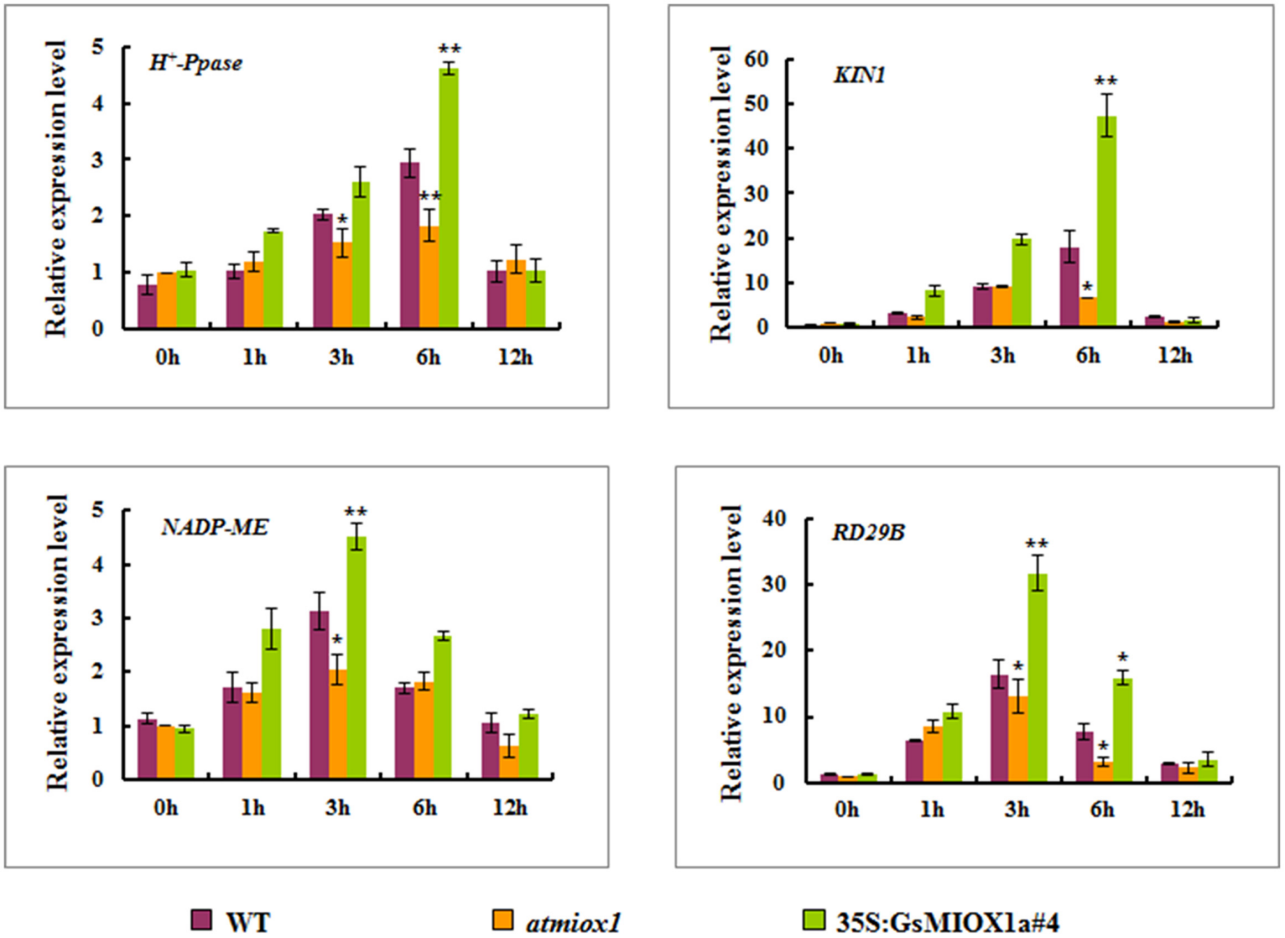
Expression of marker genes in the WT, *atmiox1* and *GsMIOX1a* OX seedlings under alkaline stress. (A) *H* ^*+*^-*Ppase* expression levels under alkaline stress. (B) *KIN1* expression levels under alkaline stress. (C) *NADP-ME* expression levels under alkaline stress. (D) *RD29B* expression levels under alkaline stress. To explore the transcript levels of stress-responsive genes, 2-week-old WT, *atmiox1* and OX seedlings (line #4) were treated with 1/2 MS solution containing 50 mM NaHCO_3_ (pH 8.5) for 0, 1, 3, 6, and 12 h. The relative transcript levels were determined by quantitative real-time PCR. The *AtActin2* gene served as an internal reference, and transcript levels were normalized to WT at 0 h. The values represent the means of three independent biological replicates and three technological replicates for each. One or two stars correspond to p<0.05 and p<0.01, respectively.

To evaluate the mechanism of the improved alkaline tolerance conferred by *GsMIOX1a* OX, the transcript levels of various stress-related marker genes, including *H* ^*+*^-*Ppase*, *NADP-ME*, *KIN1* and *RD29B*, were analyzed under normal and alkaline stress conditions in the WT, *atmiox1* and OX plants. *H* ^*+*^-*Ppase* excludes H^+^ ions via H^+^ pumps located in the plasmalemma and tonoplast of plant cells [34], and *NADP-ME* balances malic acid levels [35]. Thus, both of these proteins function in adjusting the pH of the cytoplasm (pHc) and aid the mitigation of the high pH caused by alkaline stress. *KIN1* and *RD29B* have long been proposed to play roles in various stress responses [36, 37], including alkaline stress. Quantitative real-time PCR analysis indicated that all of the selected marker genes were significantly induced by NaHCO_3_ in the *GsMIOX1a* OX lines but significantly decreased in the *atmiox1* line. In all lines, *H* ^*+*^-*Ppase* and *KIN1* expression levels peaked at 6 h, whereas *NADP-ME* and *RD29B* peaked at 3 h (Fig 6). These results also imply that *GsMIOX1a* OX promotes the accumulation of stress-responsive genes transcripts, which might be helpful for alkaline tolerance.

## Discussion

Inositol is ubiquitous across the biological kingdom and is an essential component of eukaryotic cells [38]. Plants maintain an inositol pool at a basal level throughout their life cycle, and MIOX is used to control the metabolite level of *myo-*inositol in plants. In a few stress-tolerant plants, MIOX plays a role in the response to environmental stresses. These stress-tolerant properties are suggested to arise due to adaptive changes in their coding sequences, depending on the environment in which the plants live. For example, upland rice, which is normally grown in well-drained soil with low water-holding capacity and no surface water accumulation, is reported to encode drought-tolerant MIOX, whereas lowland rice, which is very susceptible to drought stress, encodes drought-sensitive MIOX [22]. In this study, we used *G*. *soja* 07256 because it is a relatively alkaline-tolerant plant that can germinate and set seeds in sodic soil at pH 9.02 and can survive in nutrient solutions containing 50 mM NaHCO_3_ [8]. Because of this adaptation to harsh environmental conditions, *G*. *soja* 07256 is rich in resistance genes for alkaline stress tolerance.

Our lab recently analyzed RNA-seq data from roots of *G*. *soja* 07256 subjected to alkaline stress sampled at different time points (0, 1, 3, 6, 12 and 24 h). We observed that *GsMIOX1a* was strongly up-regulated among the five members of the MIOX family (Figure A in S1 File). Although the *GsMIOX2a* gene appears to be induced more dramatically than *GsMIOX1a*, functional verification is ongoing. As a result, we focused on *GsMIOX1a*. In this study, the experimental data suggested that *GsMIOX1a* is an early alkaline stress-responsive gene in *G*. *soja* 07256 (Fig 2A), consistent with the RNA-seq data. The response of *GsMIOX1a* to alkaline stress was further confirmed in different soybean varieties (Fig 2B). As expected, the highest expression change was observed in *G*. *soja* 07256. The different expressions of *MIOX1a* in different soybean varieties suggest the importance of *GsMIOX1a* in the plant response to alkaline stress. Similarly, in upland rice (*Oryza sativa* L. cv. IRAT109) not in lowland rice, *OsMIOX* is induced by drought, H_2_O_2_, salt, cold and abscisic acid [22]. No *MIOX* transcript analyses were conducted under abiotic stresses in stress-sensitive plant species, such as *Arabidopsis thaliana*, but *AtMIOX4* OX *Arabidopsis* lines did not exhibit a significant phenotype under different concentrations of NaCl and sorbitol, thus suggesting a null function for *MIOX4* under stress conditions [20]. These reports indicate that stress-induced expression of the *MIOX* gene appears to be species specific as well as dependent on the stress-responsive nature of the species.

Earlier studies have demonstrated that *MIOX* genes are differentially expressed in different organs of certain plant species, such as *Arabidopsis thaliana* [39] and rice [22]. In this study, *GsMIOX1a* transcripts were observed in all examined tissues, and the most abundant levels were noted in flowers (Fig 2C). This result suggests that the *GsMIOX1a* gene is differentially regulated in different organs to coordinate inositol metabolism. Subsequent experimental data suggested that the *GsMIOX1a* gene was functional in the *Arabidopsis* plant. In addition, *GsMIOX1a* gene expression in *Arabidopsis* plants improved the tolerance to alkaline stress in the germination and mature stages, whereas *atmiox1* impaired tolerance at both stages (Figs 4 and 5A). Alkalinity triggers osmotic stress; therefore, many plants accumulate proline as an osmoprotectant to resist detrimental effects on cellular components [40]. Proline protects plants from stress through different mechanisms, including contribution to detoxification of reactive oxygen species, cell turgor pressure maintenance, stabilization of enzymes/proteins and scavenging free radicals, to counteract the effects of osmotic stress [41]. Moreover, alkaline conditions also promote a rapid accumulation of ROS, which can cause cell damage and even death. Scavenging excessive ROS can avoid or alleviate the damage induced by stress to plant metabolism, enhancing the tolerance for alkalinity [42]. Our results suggested that the stress-induced growth inhibition of *atmiox1* plants was associated with a reduced level of proline and POD activity, whereas the OX lines that accumulated more proline and had higher POD activity displayed enhanced growth responses under alkaline stress conditions. However, when plants were grown in a normal environment, we did not observe any apparent advantages for the OX lines (Fig 5B and 5C). These findings corroborate the results of J. Duan [22], who reported enhanced drought tolerance in rice (*Oryza sativa* L.) overexpressing *OsMIOX* (MIOX coding gene from upland rice). This tolerance was attributed to increased ROS scavenging enzyme activity and proline content in OX plants. Based on our results, we hypothesize that the *GsMIOX1a* OX plants exhibit increased alkaline stress tolerance by regulating downstream metabolism and accumulating more proline with improved POD activity, thereby counterbalancing excessive ROS, which may account for increased alkaline tolerance. In addition, proline can scavenge ROS via an unclear mechanism [43], indicating that the higher levels of proline in OX plants may also scavenge ROS.

The main product of the MIOX pathway is UDP-GlcA. UDP-GlcA provides approximately 50% of the cell wall biomass and represents a key metabolite for the nucleotide sugar inter-conversion pathways [44–46]. UDP-GlcA is important for *Arabidopsis* root hair elongation [47], and the down-regulation of UDP-GlcA biosynthesis leads to swollen plant cell walls and severe developmental defects [48]. We observed enhanced tolerance towards alkaline stress in *GsMIOX1a* OX plants, and we hypothesized that *GsMIOX1a* OX improves UDP-GlcA levels, enhances root hair elongation and results in enhanced adaption to alkaline stress. However, further analysis is required to understand the MIOX-mediated cellular processes underlying alkaline stress tolerance.

To illustrate the mechanisms of altered alkaline stress tolerance conferred by the *GsMIOX1a* OX lines and *atmiox1*, we assessed the effects of *GsMIOX1a* OX and a*tmiox1* on the transcript levels of various abiotic stress-inducible marker genes (*H* ^*+*^-*Ppase*, *NADP-ME*, *KIN1* and *RD29B*). Under alkaline stress, all selected marker genes exhibited increased expression levels in the OX plants compared with WT plants, whereas the expression of these genes was decreased in knockout mutant (Fig 6). *KIN1* and *RD29B* play important roles in adjusting the physiological conditions in plant cells [49]. The increased expression of these stress-inducible genes under alkaline stress conditions might contribute to improved alkaline tolerance in OX plants. *H* ^*+*^-*Ppase* and *NADP-ME*, which function in intracellular pH regulation and acidification of the cytoplasm under stress, are induced by alkaline stress [50, 51]. Therefore, the enhanced expression of *H* ^*+*^-*Ppase* and *NADP-ME* may counterbalance the high pH and increase alkaline tolerance. However, we also should note that *NADP-ME* and *RD29B* were also induced in *atmiox1*. This induction may be attributed to the functional redundancy of the four *MIOX* genes in *Arabidopsis*. Therefore, the null function of a gene due to its mutation may be complemented by contributions from the other genes, and thus the induction is continuous.

The major plant AsA synthesis pathway is thought to occur via guanosine diphosphate-mannose (GDP-Man) [52, 53]. However, the presence of other AsA pathways in plants is controversial. Agius et al. [54] provided molecular evidence of the use of D-GlcUA as a precursor in AsA biosynthesis in a study in which the strawberry gene for D-GalUA reductase was overexpressed in *Arabidopsis*. Two studies have also reported the additional role of D-GlcUA in plants as a potential precursor for AsA [19, 33]. Based on these studies, an animal-like alternative biosynthesis route for AsA mediated by MIOX has been proposed. To determine if AsA biosynthesis was affected by *GsMIOX1a* OX or *atmiox1* mutant, AsA levels in WT, *atmiox1* and OX lines were measured. No differences in AsA levels were observed among WT, *atmiox1* and OX plants under normal and alkaline stress conditions (Fig 5F and 5G), consistent with the results of previous studies [20, 21]. Therefore, a similar role of MIOX in plants and animals is questionable, and *GsMIOX1a* may not participate in AsA synthesis.

Taken together, these results suggest that the overexpression of *GsMIOX1a*, a novel gene identified in *G*. *soja* 07256, can endow *Arabidopsis* with alkaline tolerance, likely via the activation of proline biosynthesis, ROS scavenging and increased expression levels of stress-inducible marker genes. This study is the first to demonstrate that ectopic expression of MIOX improves alkaline tolerance in plants and will provide insight into salt-alkaline soil management.

## Supporting Information

S1 Fig Multiple sequence alignment of the full-length amino acid sequences of GsMIOX1a and MIOXs from other species. (TIF)

S1 File Expression levels of the five *G*. *soja* 07256 MIOX subgroup genes in plants treated with 50 mM NaHCO_3_ (pH 8.5), as determined by RNA-seq analysis. (TIF)
