# Supplementary figures and images for: Ectopic Expression of a Glycine soja myo-Inositol Oxygenase Gene (GsMIOX1a) in Arabidopsis Enhances Tolerance to Alkaline Stress
Source: PLoS One. 2015 Jun 19;10(6):e0129998. doi: 10.1371/journal.pone.0129998 (PMC4474918; doi:10.1371/journal.pone.0129998)

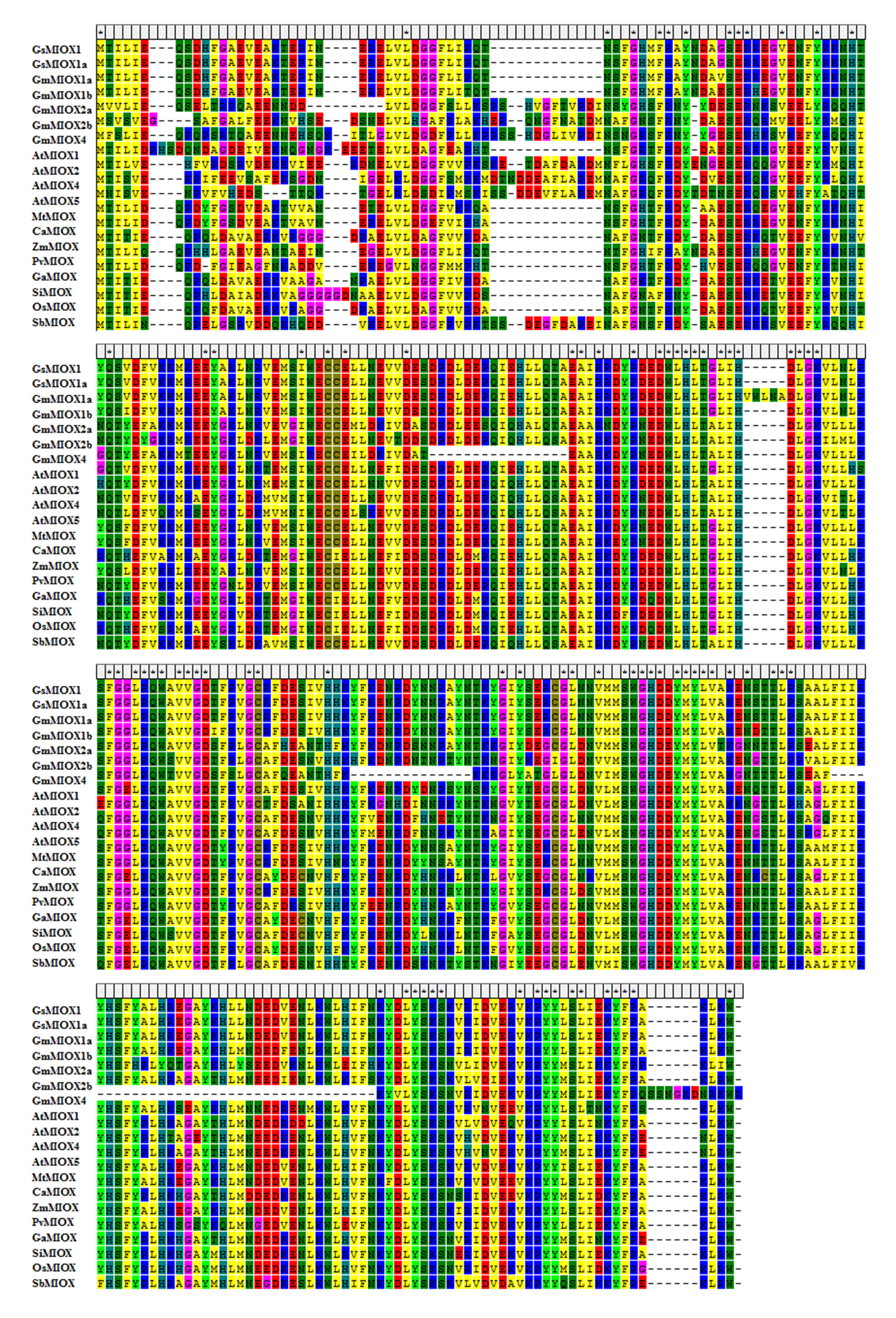

Supplement: S1 Fig — (TIF) [file pone.0129998.s001.tif]

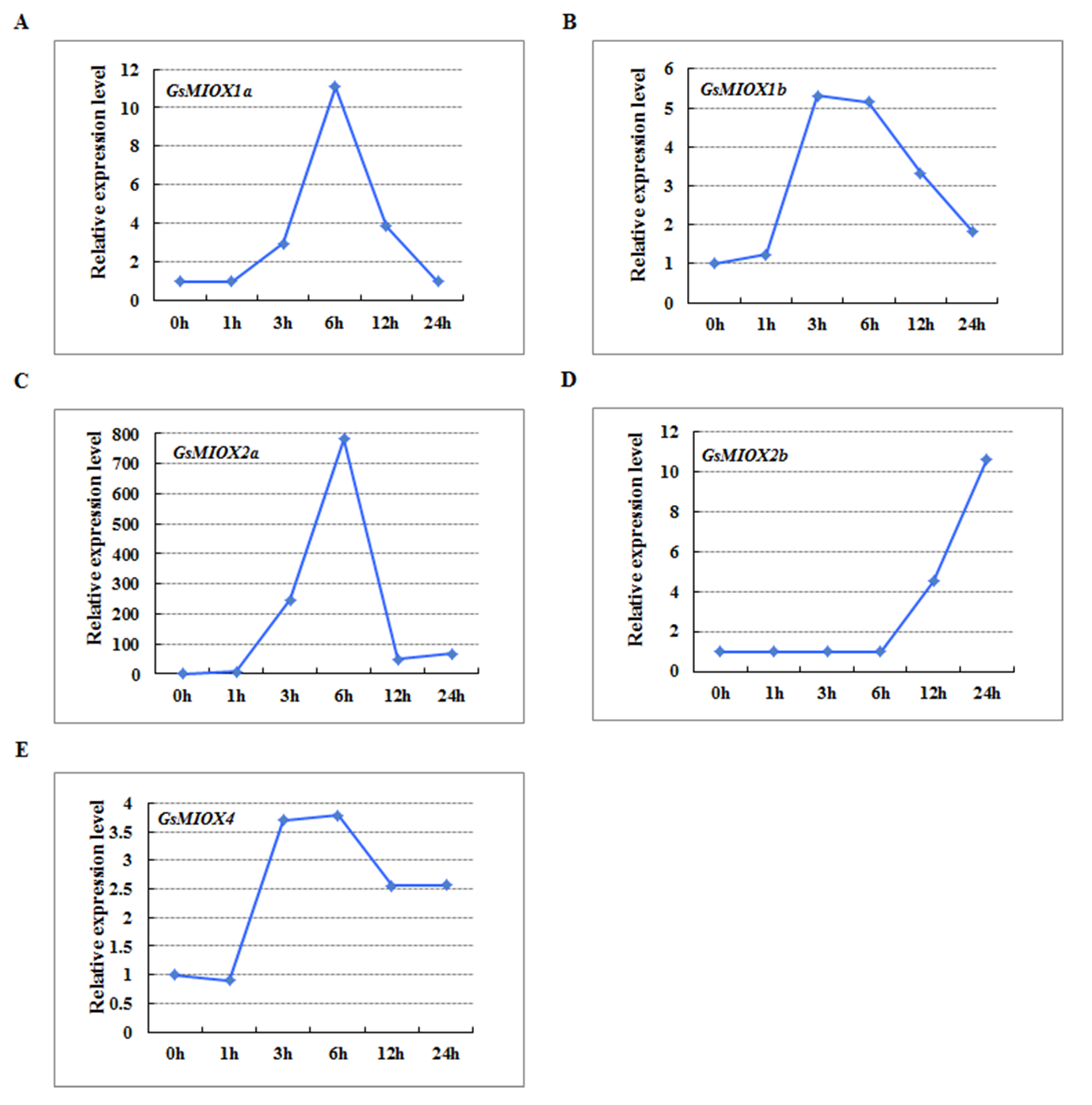

Supplement: S1 File — (TIF) [file pone.0129998.s002.tif]
